# Solution-processable electrode-material embedding in dynamically inscribed nanopatterns (SPEEDIN) for continuous fabrication of durable flexible devices

**DOI:** 10.1038/s41378-021-00307-5

**Published:** 2021-09-27

**Authors:** Wonseok Lee, Hyoungseok Chae, Dong Kyo Oh, Minyoung Lee, Hyunsoo Chun, Gyubeom Yeon, Jaewon Park, Joohoon Kim, Hongseok Youn, Junsuk Rho, Jong G. Ok

**Affiliations:** 1 Department of Mechanical and Automotive Engineering, Seoul National University of Science and Technology, Seoul, 01811 Republic of Korea; 2 Department of Mechanical Engineering, Pohang University of Science and Technology (POSTECH), Pohang, 37673 Republic of Korea; 3 Graduate Program of Energy Technology, School of Integrated Technology, Institute of Integrated Technology, Gwangju Institute of Science and Technology, Gwangju, 61005 Republic of Korea; 4 Department of Mechanical Engineering, Hanbat National University, Daejeon, 34158 Republic of Korea; 5 Department of Chemical Engineering, Pohang University of Science and Technology (POSTECH), Pohang, 37673 Republic of Korea; 6 POSCO-POSTECH-RIST Convergence Research Center for Flat Optics and Metaphotonics, Pohang, 37673 Republic of Korea

**Keywords:** Electrical and electronic engineering, Electronic devices, Structural properties, NEMS

## Abstract

A facile and scalable lithography-free fabrication technique, named solution-processable electrode-material embedding in dynamically inscribed nanopatterns (SPEEDIN), is developed to produce highly durable electronics. SPEEDIN uniquely utilizes a single continuous flow-line manufacturing process comprised of dynamic nanoinscribing and metal nanoparticle solution coating with selective embedding. Nano- and/or micro-trenches are inscribed into arbitrary polymers, and then an Ag nanoparticle solution is dispersed, soft-baked, doctor-bladed, and hard-baked to embed Ag micro- and nanowire structures into the trenches. Compared to lithographically embossed metal structures, the embedded SPEEDIN architectures can achieve higher durability with comparable optical and electrical properties and are robust and power-efficient even under extreme stresses such as scratching and bending. As one tangible application of SPEEDIN, we demonstrate a flexible metal electrode that can operate at 5 V at temperatures up to 300 °C even under the influence of harsh external stimuli. SPEEDIN can be applied to the scalable fabrication of diverse flexible devices that are reliable for heavy-duty operation in harsh environments involving high temperatures, mechanical deformations, and chemical hazards.

## Introduction

Metal nanostructures, including nanoporous metal films, self-assembled metal layers, and micro- and nanopatterned metal films, have inspired many diverse functional applications in photonics, plasmonics, and electronics^1–5^. Among these structures, metal micro- and nanopattern structures (MNPs) have provided highly accessible and widely applicable frameworks for most practical devices, including transparent electrodes, sensor platforms, and plasmonic templates^5–10^. For instance, silver (Ag) has been one of the most versatile MNP materials owing to its excellent mechanical malleability, robust electrical and thermal conductivity, and unique plasmonic and photonic characteristics^11–16^.

The fabrication of MNPs typically involves three major steps of metal deposition, lithographic patterning, and etching^17,18^. Common protocols for each step have been well established for years, such as by relying on physical vacuum deposition, photo-, e-beam, and nanoimprint lithography, wet and dry etching, and so on^19–23^. For example, sputtering is a widely used physical vapor deposition, which can manipulate optical reflectivity in the UV/Vis range with the deposited nanoparticle size, distance, shape, and interface morphology^24,25^. While effective, these conventional methods are often challenged by the increasing demand for MNPs due to their intrinsic limits on cost, time, and area^26^. To overcome these comprehensive problems, alternative nanofabrication techniques have been introduced^27–32^. However, the needs of high-temperature vacuum processing and/or plasma/chemical etching can still restrict the applicable materials, especially for flexible and organic-based devices^33,34^. In addition, such a lithographically defined MNP is typical of an ‘embossed’ structure on the substrate surface, which is prone to be damaged over time by external irritations, such as scratches and hard contacts, or internal degradations, such as cracks and delaminations^35–37^.

In these regards, a more productive and scalable methodology is needed for a facile and cost-effective fabrication of more durable MNPs on desirable flexible substrates^38–41^. More specifically, the proposed method may realize photomask- and etch-free micro- and nanopatterning and vacuum-free metalizing therein at a low temperature^42–45^. To this end, a solution-processable electrode-material embedding in dynamically inscribed nanopatterns, named SPEEDIN, is developed in this study. First, in SPEEDIN, a micro- or nanopatterned trench structure is continuously created on flexible polymers, such as polyimide (PI) substrate, with high mechanical and resistant chemical properties by dynamic nanoinscribing (DNI)^46,47^. In DNI, an edge of a patterning mold continuously inscribes the seamless micro- or nanograting pattern on various flexible substrates through a linear stroke under conformal contact, but there is no systematic DNI application on PI due to its tough machinability; also, multiple DNI strokes can be sequentially combined to readily create multidimensional patterns on PI. Then, a colloidal Ag nanoparticle solution is simultaneously coated on and doctor-bladed off the trench-patterned surface, which is annealed by slight baking to embed the Ag wires selectively inside trenches.

SPEEDIN uniquely enables the continuous and high-speed embedding of highly durable metal structures in scalable micro- and nanopatterns without resorting to masked lithography, vacuum deposition, or additional etching. Overcoming the limits and drawbacks of conventional embossed MNPs vulnerable to physical damages, SPEEDIN can achieve the highly durable metal frameworks generally applicable to heavy-duty flexible devices. As one vivid example, a flexible device that can be reliably heated up to 300 °C, even under harsh environments involving scratching, tension, bending, and twisting, is demonstrated.

## Results and discussion

**Fig. 1:**
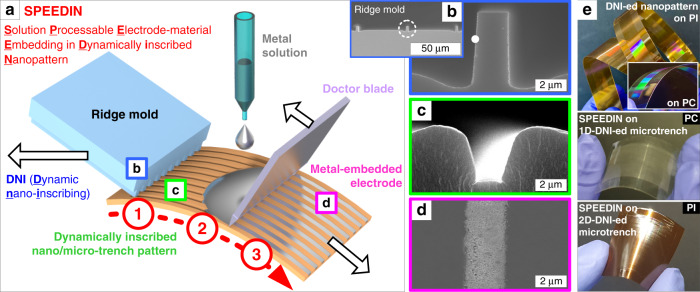
Solution-processable electrode-material embedding in dynamically inscribed nanopatterns (SPEEDIN). **a** Schematic illustration of the SPEEDIN process comprising three sequential processes: (1) DNI, (2) metal solution coating, and (3) doctor-blading. The microridge mold (**b**) can continuously inscribe the (**c**) microtrench where (**d**) the metal microwire can be selectively embedded by controlled coating, baking, and doctor-blading. **e** Examples of the seamless large-area nanopatterns continuously DNI-ed on PI (top) and PC (top inset), and the SPEEDIN-ed sample on flexible PC (middle) and PI (bottom) substrates

The SPEEDIN procedure comprises three main steps: DNI patterning, metal solution coating, and doctor-blading, all of which can be performed in a continuous and high-speed fashion (~10 mm/s) without time-consuming lithography and vacuum aids, thus making it configurable as a single flow-line manufacturing system. Figure 1a schematically illustrates such a continuous, single-stroke principle of SPEEDIN. First, a well-cleaved Si ridge mold edge (~2 µm linewidth, 5 µm height, and 50 µm period, unless otherwise noted; Fig. 1b) slides on a flexible substrate under conformal contact (with a typical force of 1–5 N) and proper heating (typically near the glass transition temperature (*T*_*g*_) of the substrate material). Here, the substrate surface undergoes continuous plastic deformation to the trench pattern (Fig. 1c) without surface degradation by the continuous inscription of the ridges in a mold edge.^46^ The substrate materials employed in this DNI patterning include polycarbonate (PC) and PI, which can be seamlessly patterned over large areas as exhibited in Fig. 1e. While the patterning depth and profile can be tailored with a critical resolution of an ~50 nm period and an ~1:1 aspect ratio by controlling the inscribing force, temperature, and speed, as systematically demonstrated elsewhere^46^. The DNI patterning temperatures for PC and PI were controlled to near their respective *T*_*g*_’s of 150 °C and 310 °C, with the contacting force set to 3 N in this study, to ensure consistent and faithful trench patterning. Notably, DNI can readily create scalable micro- and nanopatterns on the PI film that otherwise demands tricky etching work owing to its excellent mechanical and chemical durability, which makes it particularly promising for developing diverse ‘heavy-duty’ flexible MNP devices.

An aqueous colloidal solution containing Ag nanoparticles (average diameter ~100 nm) is then coated on the DNI-patterned surface, which is soft-baked, doctor-bladed, and hard-baked to leave the solidified Ag wires embedded in the trenches as shown in Fig. 1d. Figure S1 depicts the exemplary SPEEDIN procedure. Here, the tactful arrangement of the Ag embedding sequence comprising soft- and hard-baking and doctor-blading is very important for successful SPEEDIN. Figure S2 in the Supplementary Information comparatively demonstrates several control arrangements of soft-baking, hard-baking, and doctor-blading sequences for the 1D- and 2D-DNI-ed microtrench pattern surfaces. If doctor-blading is applied to the as-coated, nonbaked Ag solution, most Ag, remaining liquid, is swept away with poor embedding into the DNI-ed trench. If doctor-blading is applied late after all baking is finalized, the already-solidified Ag layer cannot be cleanly peeled off the surface, leaving a spotty Ag residue behind. A soft-baking (typically at 100 °C for 1 min) approach can change the as-coated Ag solution to a viscous concentrate with reduced solvent but still keeps it in a liquid phase. Doctor-blading at this time can selectively scrape off the top surficial Ag coating while keeping the trench-embedded Ag in place. Hard-baking (typically at 120 °C for 5 min) is then applied to fully remove the residual solvent and complete the SPEEDIN structure.

An adequate initial Ag solution coating is another essential consideration for reliable SPEEDIN. Airbrushing may be one possible candidate for conformal and scalable coating of the Ag nanoparticle solution over the DNI-ed topographic surface. However, the uncontrolled solvent vaporization concurrently occurring during airbrushing can force the Ag nanoparticles to be spread and dried all over the surface. This can disturb the clean, selective scraping-off process, which is experimentally verified as shown in Fig. S3 in the Supplementary Information. Drop-casting may be another practical choice, especially for microscale trench patterns. However, dropping an excessive amount of the Ag solution can cause difficulty for uniform and time-efficient soft-baking and is prone to wasting the overflown Ag nanoparticles upon doctor-blading. We could obtain an optimal thickness of Ag thin film by controlling the spin-coating speed depending on the microtrench width. For the typical 1–2 µm-wide trenches shown in Fig. 1, a coating speed of ~1000 rpm is reasonable for the Ag solution used in this study; too fast a coating speed (e.g., ~3000 rpm) could undesirably curtail the embedded Ag wire width due to insufficient initial coating (see Fig. S4 in the Supplementary Information for comparative results). For wider microtrench patterns, such as of an ~10 µm width, we can lower the coating speed (e.g., ~500 rpm) to facilitate the Ag solution filling into the trenches. For nanoscale trench patterns, as the effect of capillary force becomes more pronounced, the drop-casting of a controlled amount of the Ag solution under gentle agitation can be a rational method. Several exemplary SPEEDIN structures fabricated on wider microtrench and nanotrench patterns are demonstrated in Fig. S5 in the Supplementary Information.

**Fig. 2:**
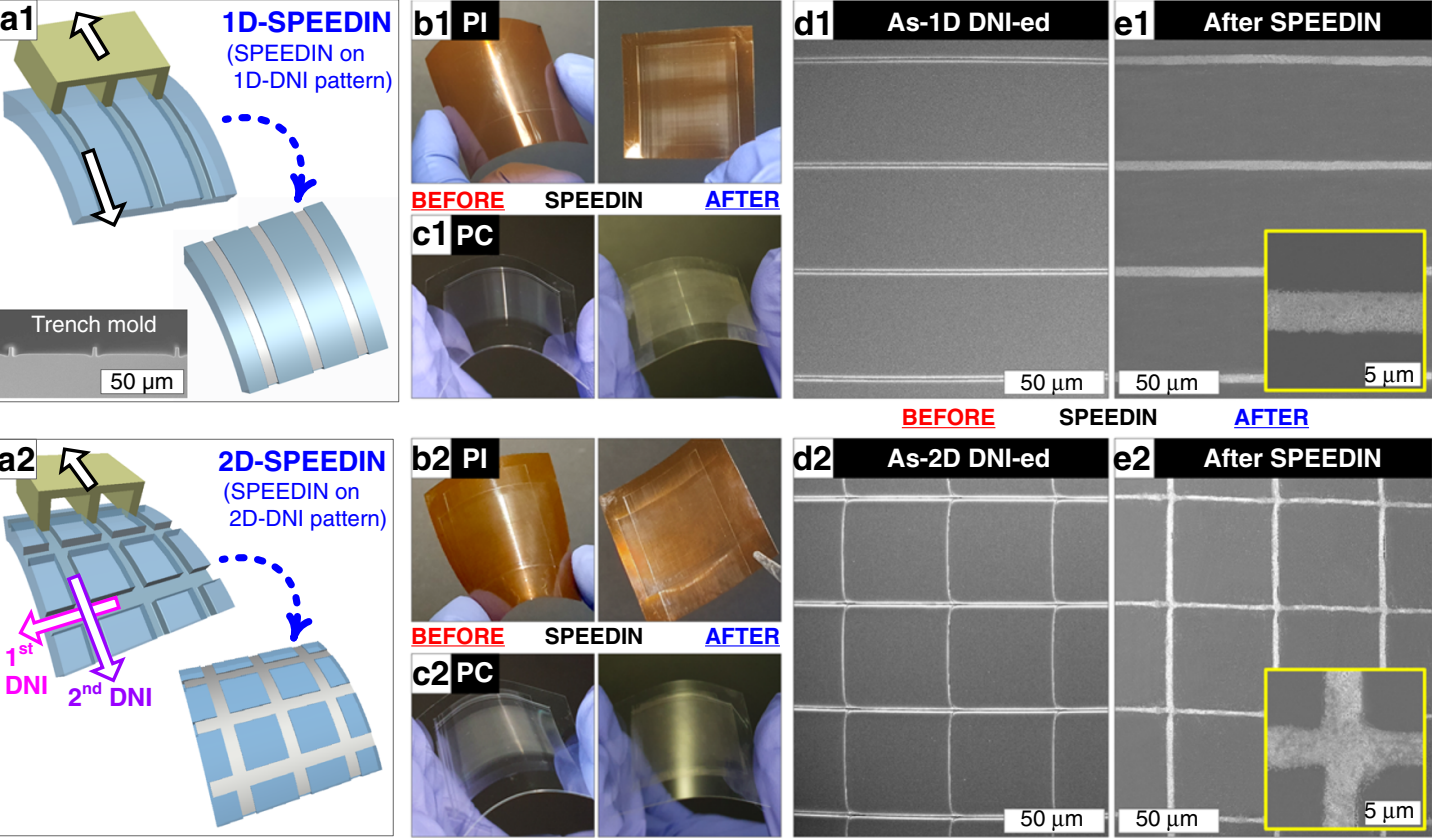
Comparative demonstration of the 1D-SPEEDIN (top row) and 2D-SPEEDIN (bottom row). **a** illustrations of DNI schemes before and after Ag embedding, (**b**) as-DNI-ed (left) and SPEEDIN-ed (right) samples on PI, (**c**) as-DNI-ed (left) and SPEEDIN-ed (right) samples on PC, and SEM images of (**d**) as-DNI-ed and (**e**) SPEEDIN-ed structures. The insets to (**e**) show the enlarged views of embedded Ag microwires

SPEEDIN can achieve the facile, scalable, and clean embedding of metal microwires in various polymer films. Figure 2 collectively demonstrates the SPEEDIN results for 1D- and 2D-DNI-ed microtrench patterns created on PI and PC substrates. The DNI patterning can be sequentially performed along multiple directions to create 1D, 2D, and further multidimensional micro- and nanopattern structures, as schematically illustrated in Fig. 2a. Notably for 2D-DNI, after the first DNI stroke was made with the contacting force of 3 N, the force during the second stroke was controlled to be ~50% (i.e., ~1.5 N) in order to avoid excessive deformation of the first-stroked DNI pattern lines. This can secure the ‘open crossing’ structure for each intersection, which is favorable for mesh-type metal nanowire fabrication by subsequent Ag solution embedding (see the inset to Fig. 2e2). The comparative optical images taken before and after performing 1D- and 2D-SPEEDIN on PI and PC are shown in Fig. 2b and c, respectively. The scanning electron microscopy (SEM) imaging for these structures (Fig. 2d and e; before and after SPEEDIN, respectively) confirms the clean, well-connected Ag microwire structures fabricated by SPEEDIN. The enlarged SEM images shown as the insets to Fig. 2e disclose that the continuous Ag lines are only inside the DNI-ed trenches, with very little Ag residue on the surrounding areas. This is also verified by energy-dispersive X-ray spectroscopy (EDX), as seen in Fig. S6 in the Supplementary Information.

**Fig. 3:**
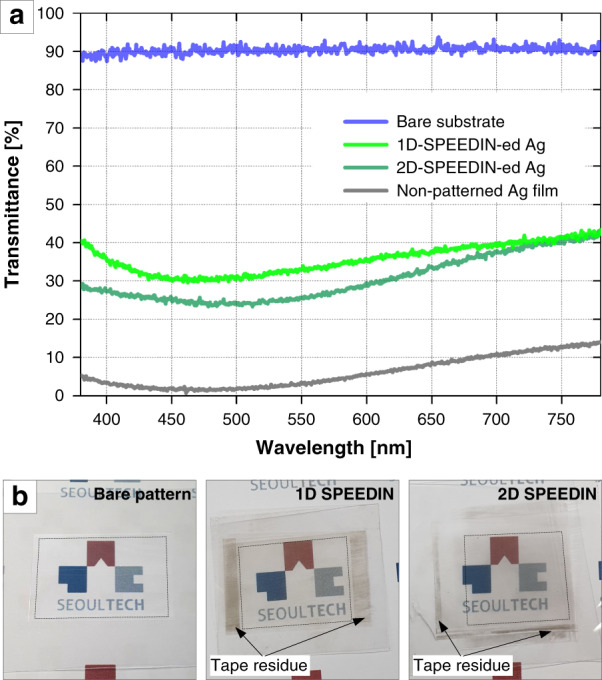
Optical properties and photographs of SPEEDIN-ed sample. **a** Optical transmittances measured for bare, 1D-SPEEDIN-ed, 2D-SPEEDIN-ed, and nonpatterned Ag-coated PC samples. **b** Optical images of the fabricated samples. The dark areas surrounding the SPEEDIN-ed areas are generated by the tape residues used for sample mounting

The SPEEDIN-ed polymer films can possess reasonable optical transmittance as well as electrical conductivity, although the embedding of Ag structures in the transparent substrates may obviously call for some compensation. Figure 3a shows the measurement results for the optical transmittances of the 1D- and 2D-SPEEDIN-ed structures formed on PC substrates (with a measured reference transmittance of ~90% with respect to air). Compared to the ~10% transmittance measured for a continuous, nonpatterned Ag layer coated on bare PC film, the 1D- and 2D-SPEEDIN-ed Ag microstructures on PC films indicate transmittances of ~30% and ~40%, respectively. The optical images of the real samples (Fig. 3b) demonstrate more vividly that these structures are transparent for clear transmission of the background images. The decrease in optical transmittance may be due to additional factors other than the Ag microwires’ light-blocking, possibly involving intrinsic scattering and surface plasmon resonance from the sparsely remaining Ag nanoparticles. Such optical characteristics of SPEEDIN-ed MNPs might be similar to those studied for the ‘embossed’ Ag MNPs conventionally relying on the photolithography-and-then-etching fabrication protocol^48,49^. Meanwhile, the sheet resistances for the 1D- and 2D-SPEEDIN structures fabricated on PI films were measured to be 26.3 and 19.8 Ω/sq on average (see Table S1 in the Supplementary Information for detailed measurement data).

**Fig. 4:**
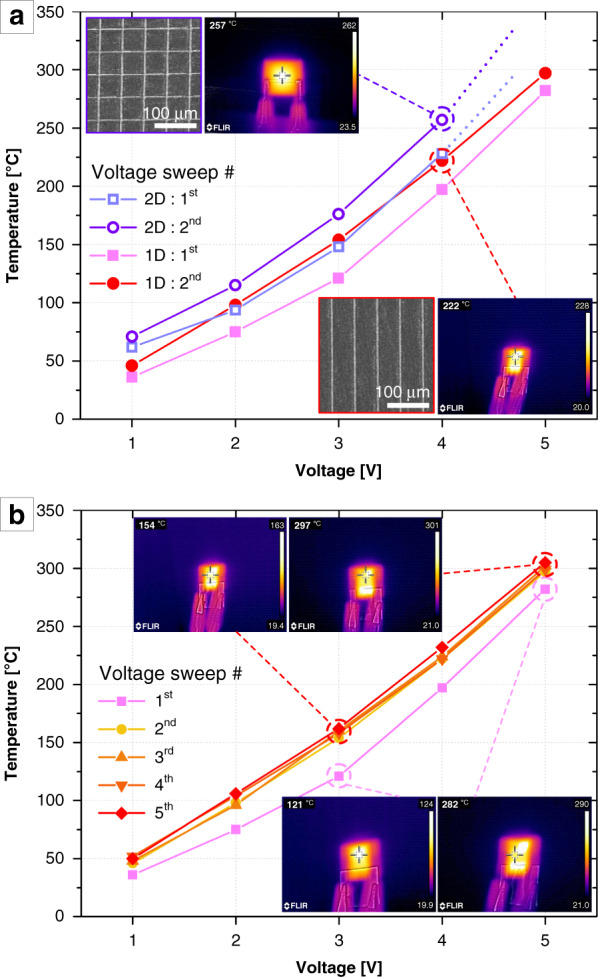
Heating devices fabricated by SPEEDIN. **a** Heating performance of the 1D- and 2D-SPEEDIN-on-PI samples, comparatively plotting the temperatures measured for the first-round and second-round operations under a voltage sweep from 1 to 5 V. The insets show the SEM images and representative IR camera images taken at the 4 V operation. **b** Five times repeated heating performance of the 1D-SPEEDIN-on-PI sample with the identical setup. The insets show the representative IR camera images taken at the 3 and 5 V operations

While these optical and electrical properties may be commonly practicable for transparent and flexible devices, the uncommon ability to the facile MNP embedding in mechanically and chemically robust substrates can allow the SPEEDIN structures to be utilized in numerous diverse applications by affording reliable operation in a harsh environment. For instance, the SPEEDIN-on-PI structures can be used as high-performance flexible and transparent joule-heaters that can achieve up to ~300 °C heating at 5 V operation voltage. Figure 4 shows the heating performances of the 1D- and 2D-SPEEDIN-on-PI samples, characterized by plotting the IR camera-measured temperature versus sweep voltage (*V*). According to Joule’s law of resistive heating: *P*~*V*^*2*^/*R* (where *R* is resistance), the power of heating *P* may be higher for more conductive electrode frameworks under the same *V* operation. Consistently, the 2D-SPEEDIN-on-PI structure can realize more power-efficient heating overall, as shown in Fig. 4a.

The cyclic operations by repeated voltage sweeps provide us with two further important findings. First, compared to the first-round heating using the as-SPEEDIN-ed structures, the follow-up rounds’ operations indicate generally improved heating efficiencies. As comparatively seen in the first- and second-round operations of the 1D- and 2D-SPEEDIN-on-PI samples (Fig. 4a), this was systematically examined through multiple times repeated operations of the 1D-SPEEDIN-on-PI sample. Figure 4b shows the heating performance for the first five-round operations, and the IR camera images taken for each measurement point are additionally provided in Fig. S7 in the Supplementary Information. Although not shown here, we additionally confirmed that the heating curve is stabilized from the initial operation, for instance, the next fifty times. We can comprehend such an interesting ‘aging’ characteristic by taking a closer look at the morphologies of the Ag wires before and after initial heating. Figure S8 comparatively shows the as-SPEEDIN-ed, never-heated Ag wire and the Ag wire ‘annealed’ with initial heating experience, along with the schematic illustrations of respective cross-sections. Initially, the as-SPEEDIN-ed Ag nanoparticles are loosely packed with nanoporous interspaces, resulting in higher contact resistance. Once these Ag nanoparticles undergo the heating cycle (i.e., thermal annealing^50,51^), they are more closely packed by merging into larger aggregations with reduced pores, as also supported by the contracted linewidth observed in the top-view SEM image. This densification of Ag nanoparticles can lead to a decrease in contact resistance and enhanced resistive joule-heating for the follow-up operations.

**Fig. 5:**
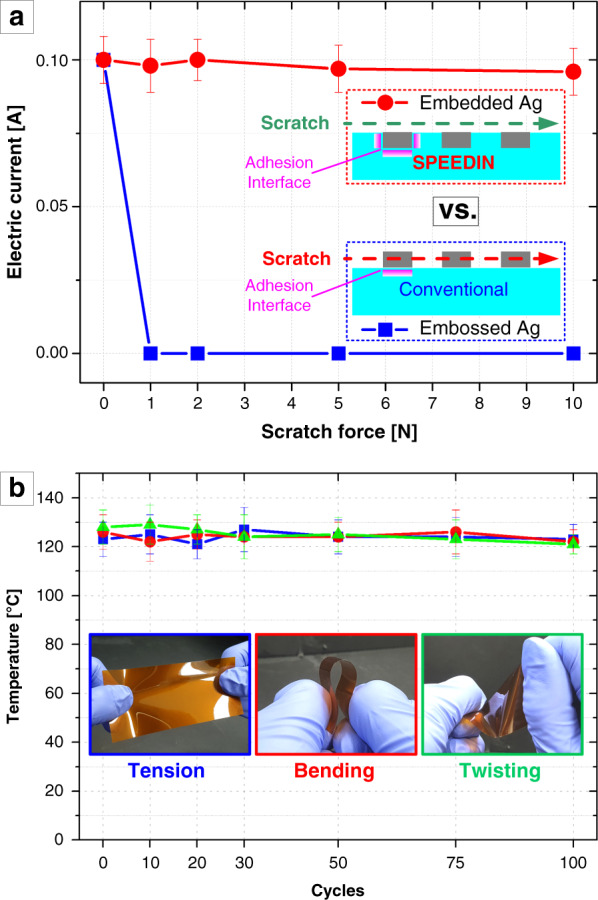
Highly durable SPEEDIN-ed metal structures. **a** Scratch test result for the SPEEDIN-on-PI sample (red dot line) and conventional photolithography-made sample (blue square line), characterized by tracking the current as the scratch force is applied. The inset images illustrate the structural schemes and adhesion interfaces between metal nanowires and substrates. **b** Durability test result for the SPEEDIN-on-PI sample, characterized by recording the heating temperature at 2.5 V operation after applying repeated instances of tension (blue line), bending (red line), and twisting (green line). The insets show the optical photographs for each disturbance application

Another crucial point discovered from the cyclic operation is that the SPEEDIN-ed structures exhibit highly consistent and reproducible heating performance upon repeated heating cycles. It is normally considered that most of the MNP-embossed and/or metallic thin-film-deposited resistive heating architectures suffer from electrode cracking and/or delamination during the repeated operations. By resolving that issue, the stable and steady cyclic operation investigated in the SPEEDIN-based heating architectures (Fig. 4) may be attributed to the enhanced adhesion between the embedded metal microwires and substrate trenches over the increased interfacial surface areas (see the inset diagrams in Fig. 5a). In addition to the robustness against those internal degradations, the SPEEDIN architectures, inherently comprising embedded wires, can provide excellent durability against external irritations such as scratches and hard contacts. Here, we conducted a simple scratch test for two types of MNP electrodes with similar wire geometries but fabricated through the different protocols: SPEEDIN and photolithography etching. Once each of the fabricated MNP samples was put on the scale and was initially set to an ~0.1 A current operation, a tweezer scratch was applied with a controlled force (read by the scale). The experimental detail of this test is described in the Experimental Section, along with the scheme in Fig. S9 in the Supplementary Information. Figure 5a shows the scratch test results for the embedded SPEEDIN and the conventional embossed MNP electrodes, characterized by plotting the electrical current versus scratch force. It can be clearly seen that the SPEEDIN-ed electrode sustains steady operation against the scratch applied with up to an ~10 N force, whereas the embossed MNP electrode is shortly disconnected right after the 1 N scratch. Although the SPEEDIN structure is sustainable beyond the ~10 N scratch, a deformation of the substrate material, such as tearing and crumpling, occurs rather than the destruction of the electrode itself. While quite simplified, this scratch test exemplifies the durability of the SPEEDIN electrodes against external mechanical stimulation compared to fragile conventional MNPs.

The durable and steadfast functioning of the SPEEDIN architecture in a highly reproducible and power-efficient fashion against both internal and external disturbances may ensure its reliability for heavy-duty flexible electronics that should endure frequent tension, bending, twisting, and so forth. As a final checkup, we examined whether the SPEEDIN device can function as a reliable and steady heater under repeated tension, bending, and twisting. Figure 5b shows the measurement result of the heating temperatures of a 1D-SPEEDIN-on-PI structure at an operating voltage of 2.5 V, after applying 0, 10, 20, 30, 50, 75, and 100 times the tension, bending, and twisting in a randomly handled manner as shown in the inset photos. It is apparent that the SPEEDIN device can maintain very stable function under those daily-life occasional disturbances. Additional characterizations involving a standard test for yielding and adhesion and a quantitative bending test with a critical radius of curvature measurement are currently underway.

## Conclusions

In summary, we have developed a facile all-solution-processable SPEEDIN methodology that enables continuous single flow-line fabrication of highly durable flexible electronic platforms without resorting to a lithography etching protocol and vacuum processing. By the tactfully controlled procedure of Ag nanoparticle solution coating, baking, and doctor-blading on multidimensional micro- and nanotrench patterns dynamically inscribed in various flexible polymers, the SPEEDIN structure can achieve durably embedded, scratch-proof MNP architectures with comparable optical transmittance as well as electrical conductivity. As one tangible example, we have demonstrated that the SPEEDIN-on-PI structures can work as high-performance flexible heaters with excellent durability and reliability. In-depth, this continuous nanoparticle embedding process can be widely applied to various dielectric materials with superior optical properties for the scalable fabrication of flexible photonic devices^52–61^. Moving forward, SPEEDIN may facilitate further practical applications, including but not limited to protective gear, heavy-duty wearable electronics, and more diverse devices operational under extreme environments involving high temperatures, severe mechanical impacts, and chemical hazards^62^.

## Materials and methods

### SPEEDIN Part I: Fabrication of micro- and nanoscale trench patterns by dynamic nanoinscribing (DNI)

First, a microridge pattern was fabricated on a 6” Si wafer by Si deep reactive ion etching. The typical microridge geometry is ~2 µm in width, 5 µm in height, and 50 µm in period (see Fig. 1b) but can be tuned to wider-period microridges, nanoridges, etc., depending on the target trench designs, as actually used for making the samples shown in Fig. S5 in the Supplementary Information. More details on the nanoscale pattern fabrication can be found elsewhere^63^. The DNI mold was then prepared by cleaving the microridge-patterned wafer along the direction perpendicular to the ridge axis, with a typical width of 1–2 cm, which can be extended for scalable DNI processing. The polymer substrates (PC; DE 1-1, CLEAR, Makrofol; PI; HN 1 mil, Kapton) were thoroughly cleaned by isopropyl alcohol (IPA) and deionized water and dried by nitrogen blowing before use. The detailed design, setup, and operation of a custom-built DNI processing system for the fabrication of 1D- and 2D-micro- and nanopatterns are described elsewhere^38,41,46^. Briefly, the ridge mold edge was mounted on a heater-attached arm and brought to a conformal contact with the substrate with a typical tilting angle of 35-40°. With a mold temperature of 150 °C for PC and 310 °C for PI and a normal contact force of 3 N maintained, the mold edge slid at a speed of 1 mm/s to continuously inscribe the micro- or nanotrench patterns on the substrate. For 2D-DNI, the second stroke was sequentially repeated along the perpendicular direction by using an ~50% reduced contact force with all the other conditions fixed.

### SPEEDIN Part II: Ag nanoparticle solution coating and embedding

The Ag nanoparticle solution (PS-004, Paru Co., Ltd.; ~100 nm average diameter) was first spin-coated on the DNI-ed substrate to achieve a specific thickness of Ag solution for an optimal doctor-blading process, typically at 1000 rpm for 1 min. The Ag solution-coated sample was then soft-baked at 100 °C for 1 min. A doctor blade typically made of an IPA-soaked fab wipe-wrapped Si piece edge was used for scraping the soft-baked Ag layer off the substrate surface at the controlled force of ~5 N, which was usually repeated five times. Finally, a hard-baked process at 120 °C for 5 min was applied to complete the SPEEDIN structure. The airbrushing of an Ag nanoparticle solution as a control experiment (see Fig. S3 in the Supplementary Information) was conducted by using a custom-designed airbrushing system whose detailed setup and operation procedure were described elsewhere^64,65^.

### Characterizations

All optical photographs were taken by using a digital camera (EOS M5, Canon). SEM imaging was performed by using a field-emission SEM (JSM-6700F, JEOL Ltd.) with an accelerating voltage of 10 kV after sputtering a thin Pt film with 2–3 nm thickness to prevent electron charging. EDX analysis was conducted during SEM imaging by using an Oxford Inca EDX system equipped with the same instrument. The optical transmittance was measured with a wavelength scanning coverage from 200 to 1025 nm by using a customized spectrometer system (Wonwoo Systems, Co., Ltd.) computer-operated by Ocean Optics software. For characterizing the electrical properties, the silver paste (ELCOAT P-100, CANS Ltd.) was first applied on both ends of the SPEEDIN-ed electrode sample. An electrical current was then recorded by using a digital multimeter (Fluke 15B+, Fluke Corp.). The heating performance was characterized by using IR cameras (FLIR E5 and E6, FLIR Systems, Inc.) under a controlled operation voltage. For preparing the embossed MNP sample for a scratch test, a photoresist microwire pattern (DNR-L300-40 (120 cP), Dongjin SemiChem, Co., Ltd.) was photolithographically defined (Karl Suss MA6) on an identically spin-coated Ag film, followed by wet etching of the exposed Ag (Etchant Type A, Transene Company, Inc.), PR strip (acetone), and IPA rinsing. After putting the sample (either the embossed or SPEEDIN-ed MNP) on a digital scale (Dretec KS-514WT), a tweezer tip was stroked across the microwire line axes while the force and current were read by the scale and DC power supply (Zhaoxin RXN-305D), respectively. The scratch test was sequentially repeated from 1 to 10 N using one sample. Figure S9 in the Supplementary Information shows photos of the described scratch-proof test. All datapoints presented with error bars in the plots denote the averaged values after repeating measurements three to five times to ensure reliability and accuracy.

## Supplementary information

Supplementary Information
