# Supplementary material for: Solution-processable electrode-material embedding in dynamically inscribed nanopatterns (SPEEDIN) for continuous fabrication of durable flexible devices
Source: Microsyst Nanoeng. 2021 Sep 27;7:74. doi: 10.1038/s41378-021-00307-5 (PMC8473567; doi:10.1038/s41378-021-00307-5)
Supplement: Supplementary file 1 — Supplementary Information [file 41378_2021_307_MOESM1_ESM.pdf]

Supplementary Information

Solution-processable electrode-material embedding in dynamically inscribed nanopatterns (SPEEDIN) for continuous fabrication of durable flexible devices

Wonseok Lee[1,†], Hyoungseok Chae[1,†], Dong Kyo Oh[2,†], Minyoung Lee[1], Hyunsoo Chun[3], Gyubeom Yeon[1], Jaewon Park[1], Joohoon Kim[2], Hongseok Youn[4], Junsuk Rho[2,5,6]*, and Jong G. Ok[1]*

[1]Department of Mechanical and Automotive Engineering, Seoul National University of Science and Technology, Seoul 01811, Republic of Korea

[2]Department of Mechanical Engineering, Pohang University of Science and Technology (POSTECH), Pohang 37673, Republic of Korea

[3]Graduate Program of Energy Technology, School of Integrated Technology, Institute of Integrated Technology, Gwangju Institute of Science and Technology, Gwangju 61005, Republic of Korea

[4]Department of Mechanical Engineering, Hanbat National University, Daejeon 34158, Republic of Korea

[5]Department of Chemical Engineering, Pohang University of Science and Technology (POSTECH), Pohang 37673, Republic of Korea

[6]POSCO-POSTECH-RIST Convergence Research Center for Flat Optics and Metaphotonics, Pohang 37673, Republic of Korea

†W. Lee, H. Chae, and D. K. Oh contributed equally to this work.

*E-mail: jsrho@postech.ac.kr (J.R.)

*E-mail: jgok@seoultech.ac.kr (J.G.O)

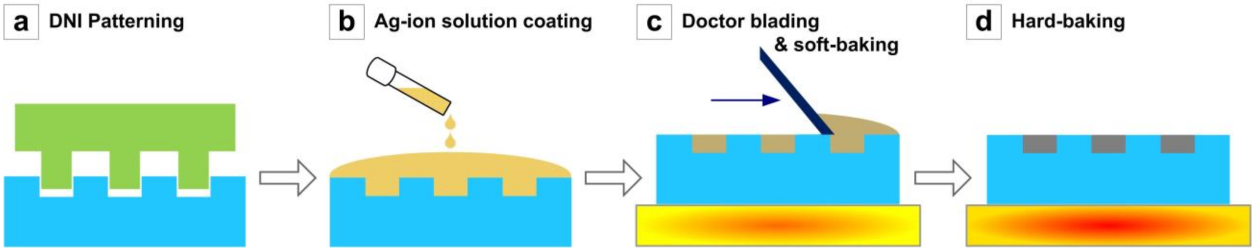

Figure S1. Exemplary SPEEDIN procedure. (a) DNI patterning, (b) Ag nanoparticle-based metal solution coating, (c) soft-baking, followed by doctor-blading, and (d) hard-baking.

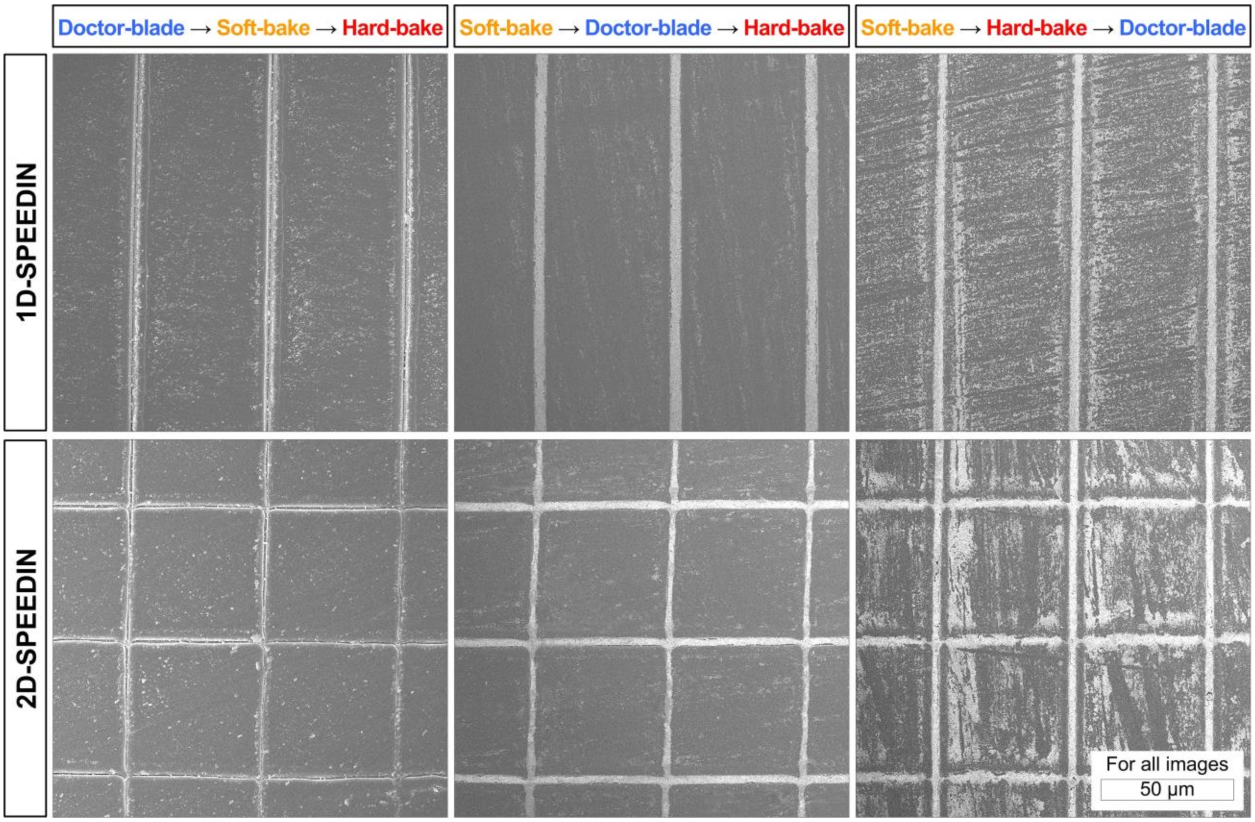

Figure S2. Optimization of SPEEDIN process. SEM imaging results of several control arrangements of soft-baking, hard-baking, and doctor-blading sequences during the Ag embedding in the microtrench patterns fabricated by 1D-DNI (upper row) and 2D-DNI (lower row). If doctor-blading is applied before any baking (left column), most Ag nanoparticles are simply swept away. If doctor-blading is applied later all baking steps (right column), the already-solidified Ag layer cannot be cleanly scraped off. The middle column shows an optimal SPEEDIN process as discussed in the main text.

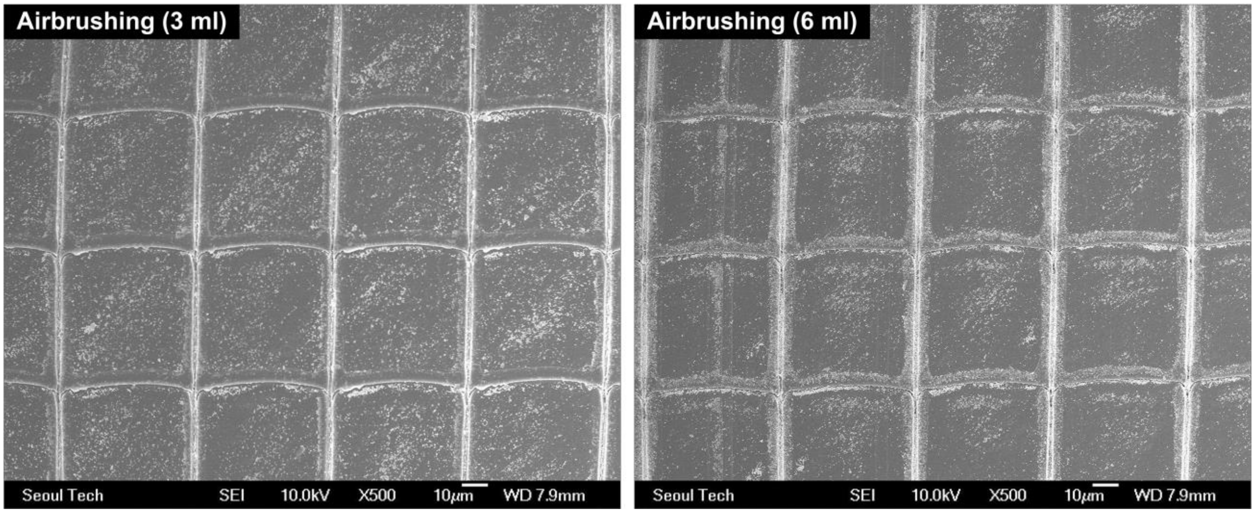

Figure S3. Optimization of Airbrushing process. SEM imaging results of control experiments for the Ag layer coating by using the 3 ml (left) and 6 ml (right) airbrushing of the Ag nanoparticle solution.

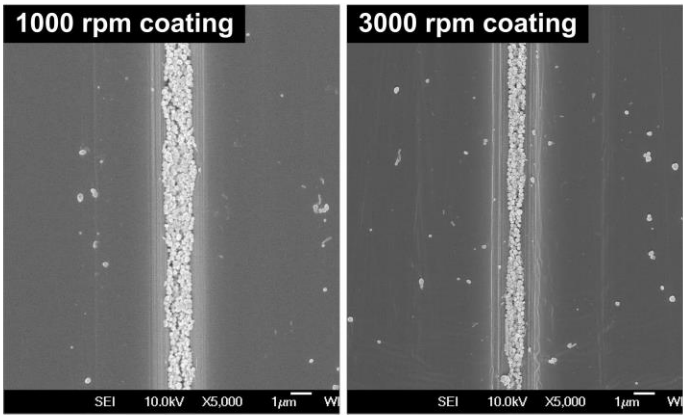

Figure S4. Optimization of spin-coating process. SEM images of the Ag wires SPEEDIN-ed from two different initial Ag layers spin-coated at 1000 rpm (left) and 3000 rpm (right).

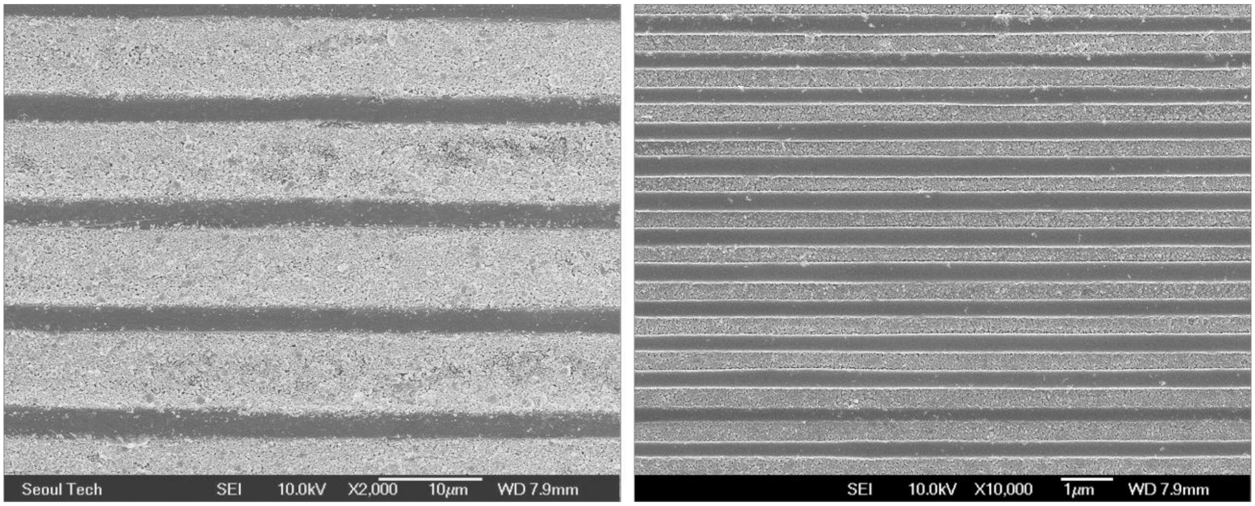

Figure S5. Comparison of SPEEDIN according to the trench period. Exemplary SPEEDIN structures fabricated on the ~10 μm-wide microtrenches (left) and the 700 nm-period nanotrenches (right).

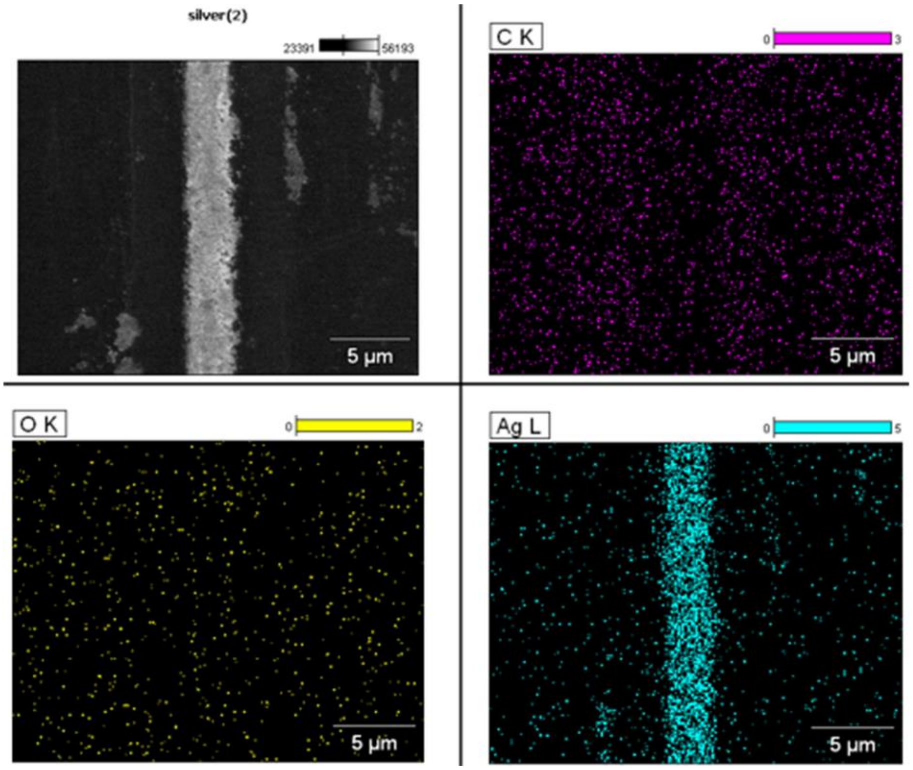

Figure S6. Analysis of constituent particles in trenches. Result of EDX analysis performed on the SPEEDIN-ed Ag wire during the SEM imaging (upper left). Uniform dispersions of C (upper right) and O (lower left) may be from the background substrate. Ag is found only in the trench with little residue on the surrounding area (lower right).

Table S1. Sheet resistance measurement data for 1D- and 2D-SPEEDIN structures fabricated on PI films.

Sample type & area (width × length [cm^2])	Measured resistance (5 times repeated) [Ω]					Average resistance [Ω]	Sheet resistance [Ω/□]
	#1	#2	#3	#4	#5		
1D SPEEDIN (0.6 × 2 cm^2)	88.5	88	88.5	87.2	86.6	87.76	26.328
2D-SPEEDIN (0.8 × 2 cm^2)	49.3	49.2	50	49.5	49.1	49.42	19.768

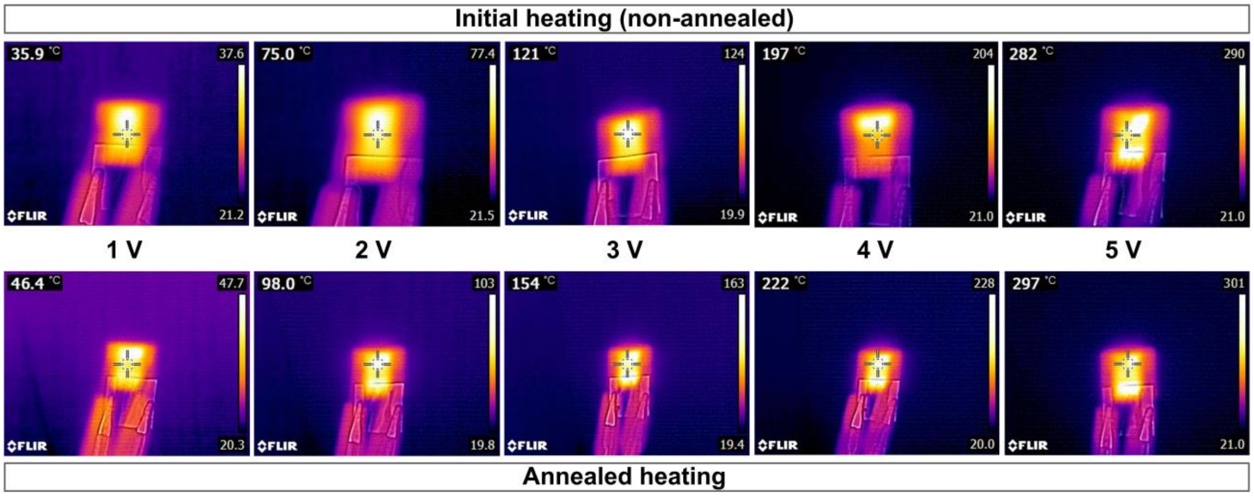

Figure S7. Measurement of heating performance. IR camera imaging results of the 1D-SPEEDIN-on-PI sample under the voltage swept from 1 to 5 V, taken for the first-round sweep (top row) and the follow-up round sweep (bottom row). The measured temperature values are plotted in Figure 5 in the main text.

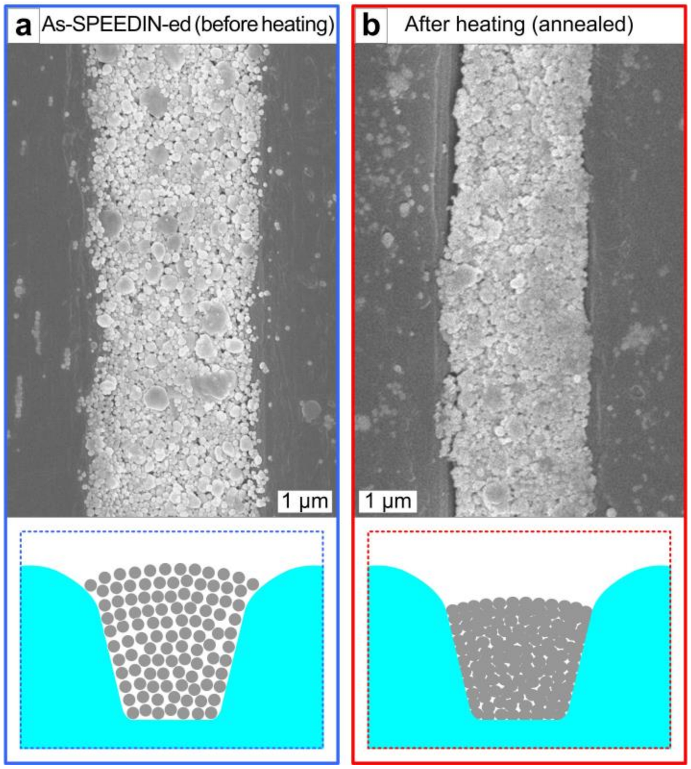

Figure S8. Observation of Ag Agglomeration. Top-view SEM images (top) and schematic cross-sectional images of the (a) as-SPEEDIN-ed microwire and (b) microwire annealed by several cycles of heating.

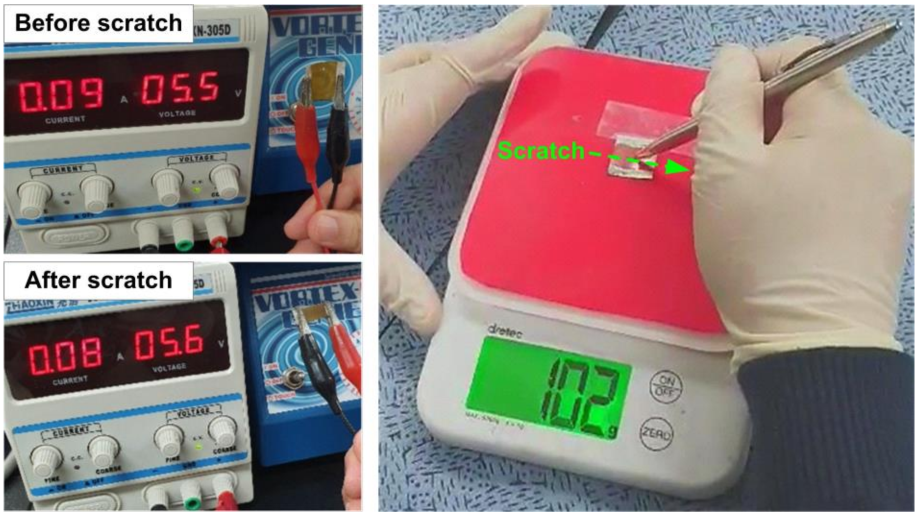

Figure S9. Scratch-proof test of the SPEEDIN-ed sample. Optical photographs demonstrating the scratch test for the SPEEDIN sample. The DC power supply (left) is connected to the sample that is put on the scale (right). While the tweezer scratch is applied at a controlled force monitored by the scale, the current is read by the DC power supply.
